# A Culture-Based ID of Micromycetes on the Wing Membranes of Greater Mouse-Eared Bats (*Myotis myotis*) from the “Nietoperek” Site (Poland)

**DOI:** 10.3390/ani10081337

**Published:** 2020-08-03

**Authors:** Rafał Ogórek, Klaudia Kurczaba, Magdalena Cal, Grzegorz Apoznański, Tomasz Kokurewicz

**Affiliations:** 1 Department of Mycology and Genetics, Institute of Genetics and Microbiology, University of Wrocław, Przybyszewskiego Street 63-77, 51-148 Wrocław, Poland; 289395@uwr.edu.pl (K.K.); magdalena.cal@uwr.edu.pl (M.C.); 2 Department of Vertebrate Ecology and Paleontology, Institute of Biology, Wrocław University of Environmental and Life Sciences, Kożuchowska Street 5b, 51-631 Wrocław, Poland; apoznanski.grzegorz@gmail.com (G.A.); tomasz.kokurewicz@upwr.edu.pl (T.K.)

**Keywords:** fungi, *Myotis*, wing membranes, hibernation, “Nietoperek” bat reserve

## Abstract

**Simple Summary:**

This study reports the colonization by fungi of the wing membranes of the female greater mouse-eared bat (*Myotis myotis*) during spring emergence from the “Nietoperek” underground hibernation site. Overall, we isolated 17 different fungal species and the most commonly isolated was *Penicillium chrysogenum*—the cosmopolitan species. Some fungal species may be pathogens of mammals, including bats. However, taking into account habitat preferences and the life cycle of bats, it can be assumed that some fungi were accidentally obtained from the surface of vegetation during early spring activity. Therefore, in the near future, we want to study the mycobiota of other bat species because they could be pathogens or part of the normal microbiome.

**Abstract:**

Bats play important functions in ecosystems and many of them are threatened with extinction. Thus, the monitoring of the health status and prevention of diseases seem to be important aspects of welfare and conservation of these mammals. The main goal of the study was the identification of culturable fungal species colonizing the wing membranes of female greater mouse-eared bat (*Myotis myotis*) during spring emergence from the “Nietoperek” underground hibernation site by the use of genetic and phenotypic analyses. The study site is situated in Western Poland (52°25′ N, 15°32′ E) and is ranked within the top 10 largest hibernation sites in the European Union. The number of hibernating bats in the winter exceeds 39,000 individuals of 12 species, with *M. myotis* being the most common one. The wing membranes of *M. myotis* were sampled using sterile swabs wetted in physiological saline (0.85% NaCl). Potato dextrose agar (PDA) plates were incubated in the dark at 8, 24 and 36 ± 1 °C for 3 up to 42 days. All fungi isolated from the surface of wing membranes were assigned to 17 distinct fungal isolates belonging to 17 fungal species. *Penicillium chrysogenum* was the most frequently isolated species. Some of these fungal species might have a pathogenic potential for bats and other mammals. However, taking into account habitat preferences and the life cycle of bats, it can be assumed that some fungi were accidentally obtained from the surface of vegetation during early spring activity. Moreover, *Pseudogymnoascus destructans* (Pd)—the causative agent of the White Nose Syndrome (WNS)—was not found during testing, despite it was found very often in *M. myotis* during previous studies in this same location.

## 1. Introduction

Bats (*Chiroptera*), inhabiting all continents except Antarctica, with more than 1400 species described so far, are the second most numerous order among all mammals [1,2]. Most species are insectivorous and serve an important role in the reduction of insect abundance, including agricultural and forest pests, and species harmful for humans such as mosquitoes (*Culicidae*) and biting midges (*Simuliidae* and *Ceratopogonidae*) [3], although in the tropics many bats developed different feeding strategies and serve as plant pollinators and seed dispersers [4]. Almost half of bats are vulnerable (VU), considered endangered (EN), near threatened (NT) or critically endangered (CR) and are listed in the IUCN Red List of Endangered Species [5,6].

In autumn, insectivorous bats inhabiting temperate zones are looking for overwintering refugia as a place of hibernation to survive adverse environmental conditions i.e., temperature drop and reduced insect availability. Selection of suitable hibernation sites, such as caves and mines, is crucial for overwinter survival [7]. Bat hibernacula need to meet specific microclimatic conditions that can sustain the hibernation cycle of bats. Some species are declining the average hibernation temperature in course of winter season to slow down the use of energy reserves accumulated in fat [8]. At the “Nietoperek” Natura 2000 site, the median temperature is 8.7 °C, (min-max 6.1–9.9 °C) and humidity varies between 77.5–100.0% [9]. It should be mentioned that temperature and humidity are among the most important factors affecting the survival of fungi in the environment [10,11]. An additional factor in determining the occurrence of fungi in underground sites are the neighboring external environment, especially local flora, the geographical location, and availability of organic matter [12,13,14]. However, it should be mentioned that low annual temperature as well as low humidity increased prevalence of microfungi on bat ectoparasites [15].

Furthermore, the organic matter is scarce in the underground, therefore, bats serve an important role for fungi survival as hosts and producers of organic waste, such as guano and dead individuals [9]. Additionally, their annual visits in the underground system sustain a stable source for external spores enriching fungi flora [9,13]. Simultaneously, during hibernation season, bats are periodically changing locations within the hibernaculum [16]. Towards the end of the winter season, some species, such as the greater mouse-eared bat (*Myotis myotis*), tend to form large clusters creating an opportunity for fungi to spread between individuals [9].

In Europe, the highest species diversity can be observed in the Mediterranean region however, due to mild winters and the overall rise in annual temperature, many species have begun to expand their ranges to the north [17,18,19,20,21,22]. The range expansion may lead to transmission of microorganisms previously unobserved in colder regions of Europe including fungi with pathogenic potential [23,24,25,26,27]. Despite the common occurrence of bats in Europe, many aspects of biology and ecology remain insufficiently studied. One concerns the characterization of the fungal community on the wing membranes of Central-European bats such as *M. myotis* [28,29]. Therefore, the number of bats and factors determining their health (including microorganisms) should be monitored in order to protect these endangered mammals [30,31].

Most of the mycological studies of the fur and skin of bats are related to their hibernation sites. For example, Johnson et al. [32] isolated the psychrophilic and psychrotolerant fungal flora of the wing membranes of hibernating bats in USA (Illinois and Indiana) such as Indiana bat (*Myotis sodalis*), tricolored bat (*Pipistrellus subflavus*), and northern myotis (*Myotis septentrionalis*). In turn, Vanderwolf et al. [25,33] and Malloch et al. [34] studied fungi associated with hibernating *P. subflavus* and little brown bat (*Myotis lucifugus*) and *M. septentrionalis* in Canada. However, most research on bat specific external fungi from temperate regions revolves around *Pseudogymnoascus destructans* (Pd) [35]. Pd causes the disease known as the White Nose Syndrome (WNS), which is a recently emerged wildlife disease that has decimated bats in North America [36,37,38,39,40,41]. Currently, the occurrence of Pd has also been found in fifteen European countries (Austria, Belgium, Switzerland, Czech Republic, Germany, Denmark, Estonia, France, Hungary, Netherlands, Poland, Romania, Slovakia, Turkey and Ukraine), but the mass deaths of the bats was not observed [9,20,23,24].

Little is known about external fungi colonizing the wing membranes of *M. myotis* during spring emergence from underground hibernation sites. The main goal of this study was to identify culturable fungal species from wing membranes of female *M. myotis* bats using genetic and phenotypic analysis, as the first step toward of monitoring bat health.

## 2. Materials and Methods

### 2.1. Study Area

Międzyrzecz Fortified Front (*Ostwall or Festungsfront im Oder-Warthe Bogen*) situated in Western Poland (52°25′ N, 15°32′ E) is a massive subterrain fortification complex built by the Germans in 1934–1944. Its Central Sector “Wysoka” (*Zentralabschnitt or Abschnitt Hochwalde*), where the observations were carried out, includes a system of concrete tunnels with a total length of approximately 32 km situated 20–30 m in the underground. The axis of the underground system called the “main road” is connected to the smaller side corridors leading to aboveground bunkers (*Panzerwerk*), that are access points for bats into the underground system. The “Nietoperek” bat reserve situated in Central Sector “Wysoka” is ranked among the top 10 largest hibernation sites in the European Union. The number of hibernating bats exceeds 39,000 individuals in some years. In 2007 the whole underground system with the surrounding surface area of 7377.37 ha became protected as Natura 2000 site “Nietoperek” (area code: PLH080003). Out of 12 bat species found hibernating in the “Nietoperek”, four are mentioned in Annex II of the EU Habitat Directive. More detailed descriptions of the “Nietoperek” bat reserve is given in our previous publications [9,42,43].

### 2.2. Sample Collection

**Figure 1:**
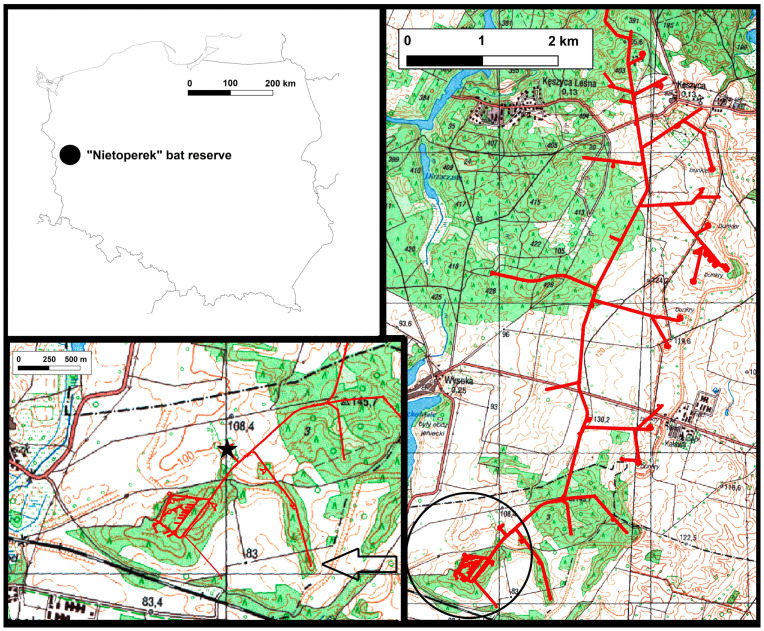
Underground corridors of Central Sector of Międzyrzecz Fortified Front in Western Poland. Netting place in the entrance to bunker Pz.W. 766 is marked by a black asterisk.

On 10th April 2016 from 21:28 and 23:31 the bats emerging from the underground fortifications of the “Nietoperek” bat reserve through the aboveground bunker Pz.W. 766 were captured by the use of two 6-m-long monofilament mist-nets (Ecotone, Poland) erected on 2.5-m-high poles (Figure 1). Using latex gloves, we determined the sex and the species using the identification key by Dietz and von Helversen [44]. Then, we collected swabs from bats wing membranes. The whole procedure lasted up to 15 min, after which the bats were released in the place of capture. Out of 18 individuals belonging to 5 species being caught, we selected the homogenous and largest sample consisting of 12 females of the greater mouse-eared bat (*M. myotis*) for analyses.

We decided to select *M. myotis* for our study because it migrates long distances between summer and winter roosts which can enhance contacts to other hosts, vectors and microorganisms as well as the microbial diversity compared to sedentary species of bats. The longest migrations of *M. myotis* from the “Nietoperek” bat reserve recorded so far exceeded 227 km [45] and cover large parts of Eastern Germany and Western Poland. Additionally, our studied species is listed in an Annex II Council Directive 92/43/EEC which places it under special protection in the European Union.

The flight membranes of *M. myotis* were sampled using sterile swabs wetted in physiological saline (0.85% NaCl) and stored in transport tubes (plastic applicator and viscose swab of 15-cm length). Every bat was sampled with two swabs from the inner surface of the flight membranes (one swab for one wing): plagiopatagium and dactylopatagia. Caution was taken to avoid cross-contamination by changing latex gloves between bats sampled. The samples were transported to the laboratory and were stored in the cold room (10 ± 0.5 °C) until mycological analysis, which was carried out within 4 days.

The observations were carried out under the license nr. WPN-I.6205.21.2016.AI provided by Regional Directorate for Environmental Protection in Gorzów Wielkopolski.

### 2.3. Isolation of Fungi from Samples

The isolation of fungi was performed using conventional culture methods. The swabs from both wing membranes of one bat were put together into one 50-mL Erlenmeyer flask containing 10 mL of sterile distilled water, and they were shaken for 20 min. Then, the biological material was plated into potato dextrose agar plates (PDA, BioMaxima, Poland) by using serial dilution isolation method. The plates were incubated in the dark at 8, 24 and 36 ± 1 °C for 3 up to 42 days (8 °C corresponds to the temperature of the hibernation place and for psychrophilic and psychrotolerant fungi; 24 °C is the optimal growth temperature of the majority of fungal species; 36 °C is the temperature oscillating with respect to the body of mammals and thermophilic species). After incubation, fungi were purified by the single spore method and were subcultured on PDA slants for morphological and molecular identification.

### 2.4. Identification of Fungi

The preliminary identification of isolated fungi was based on macro- and microscopic evaluation on the following media: Czapek Yeast Autolysate Agar (CYA, 30.0 g · L^−1^ sucrose, 15 g · L^−1^ agar, 5.0 g · L^−1^ yeast extract, 3.0 g · L^−1^ NaNO_3_, 1.0 g · L^−1^ K_2_HPO_4_, 0.5 g · L^−1^ KCl, 0.5 g · L^−1^ MgSO_4_·7H_2_O, 0.01 g · L^−1^ FeSO_4_·7H_2_O), Czapek-Dox agar (1.2% agar, BioMaxima, Poland), malt extract agar (MEA, BioMaxima, Lublin, Poland) and PDA. Plates were incubated in the dark at 15 and 20 ± 1 °C for 7–42 days. The fungi were identified according to available monographs [46,47,48,49,50,51,52,53,54,55,56,57,58]. Additionally, the phenotypes of some fungi were also compared with strains from R. Ogórek’s collection (Department of Mycology and Genetics, Institute of Genetics and Microbiology, University of Wrocław, Wrocław, Poland), which were identified using phenotypic and molecular studies, based on their internal transcribed spacer (ITS) region sequences deposited in the National Center for Biotechnology Information (NCBI, Bethesda, Rockville, MD, USA). Microscopic slides were dyed with LPCB (lactophenol cotton blue, Sigma-Aldrich). Microscopic photographs were taken with Axio Image.M1 (Zeiss, Göttingen, Germany) and macroscopic with Nikon Coolpix S3700.

To confirm species affiliation, fungal ITS region was sequenced. DNA was extracted from 21-day-old cultures on PDA medium by using Bead-Beat Micro AX Gravity (A&A Biotechnology, Gdańsk, Poland) according to the manufacturer’s instructions. Fungal rDNA was amplified using primers ITS1 (5′-TCCGTAGGTGAACCTGCGG-3′) and ITS4 (5′-TCCTCCGCTTATTGATATGC-3′) [59]. PCR was performed in a T100 Thermal Cycler (Bio-Rad, Berkeley, CA, USA), according to Ogórek et al. [60]. The PCR products were verified by electrophoretic separation on a 1.2% agarose gel and, subsequently, purified using Clean-UP (A&A Biotechnology, Gdańsk, Poland) and sequenced by the sequencing service at Macrogen Europe (Netherlands, http://dna.macrogen.com/eng/).

### 2.5. Data Analyses

The Raw sequence reads were analyzed using the BioEdit Sequence Alignment Editor (http://www.mbio.ncsu.edu/bioedit/bioedit.html) and compared with those deposited in the GenBank of the National Center for Biotechnology Information (NCBI, Bethesda, MD, USA) using the BLAST algorithm (http://www.ncbi.nlm.nih.gov/). Generated rDNA sequences were submitted to NCBI GenBank under accession numbers MN654343–MN654359.

## 3. Results

All fungi obtained from swabs were assigned to 17 distinct fungal isolates. These fungi were classified using a combination of phenotypic and molecular methods into two different phyla, 10 orders, 13 genera, including 17 different species, i.e., *Arachniotus ruber*, *Gymnascella aurantiacan* and *Arthroderma quadrifidum* (Ascomycota, Onygenales), *Aspergillus fumigatus*, *A. jensenii*, *Penicillium brevistipitatum*, *P. chrysogenum*, *P. citreonigrum* and *P. coprophilum*, (Ascomycota, *Eurotiales*), *Bartalinia robillardoides* (Ascomycota, Amphisphaeriales), *Chaetomium globosum* (Ascomycota, Sordariales), *Cladosporium sphaerospermum* (Ascomycota, Capnodiales), *Cylindrobasidium laeve* (Basidiomycota, Agaricales), *Cystobasidium ongulense* (Basidiomycota, Cystobasidiales), *Pararamichloridium caricicola* (Ascomycota, Pararamichloridiales), *Phialemonium atrogriseum* (Ascomycota, Cephalothecales) and *Scopulariopsis brevicaulis* (Ascomycota, Microascales). All fungi found in this study received internal isolate numbers from UWR_152 to UWR_167, and their ITS nucleotide sequences were submitted to GenBank under accession numbers from MN654343.1 to MN654359.1 (Table 1).

Overall, the species composition of the fungal communities inhabiting the wing membranes of female greater mouse-eared bats was different between individuals. The wing membranes of individual bats tested were colonized with 4 to 7 fungal species (Table 1). To a large extent, the species composition of fungi also depended on the incubation temperature of Petri dishes with biological material. Most fungal species (n = 16) were isolated from the tested samples using an incubation temperature at 24 ± 1 °C, and least species (n = 3) at 36 ± 1 °C. Fungal species such as *A. ruber*, *A. jensenii*, *B. robillardoides*, *C. sphaerospermum*, *C. laeve*, *P. caricicola*, *P. brevistipitatum* and *P. citreonigrum* were cultured only at 24 ± 1 °C. In turn, *G. aurantiaca* was isolated only at 8 ± 1 °C (Table 1).

**Figure 2:**
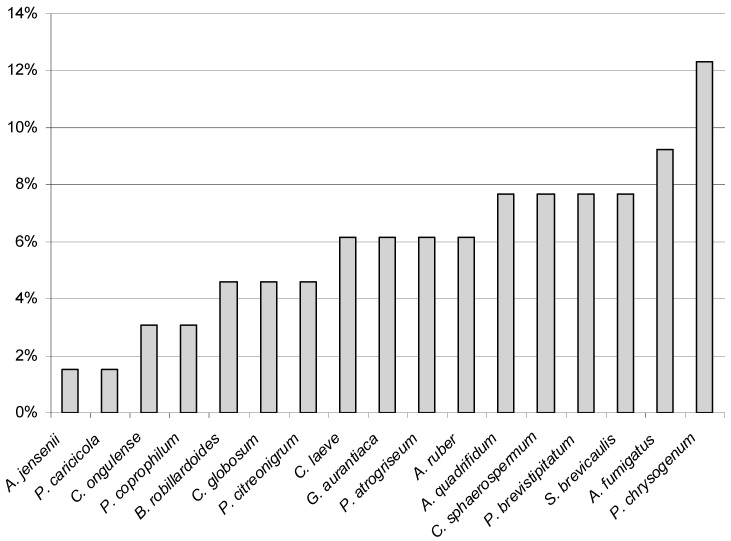
The percentage of which each species was isolated from the wing membranes of 12 female greater mouse-eared bats (*Myotis myotis*) contributed to the total from all incubation temperatures.

**Figure 3:**
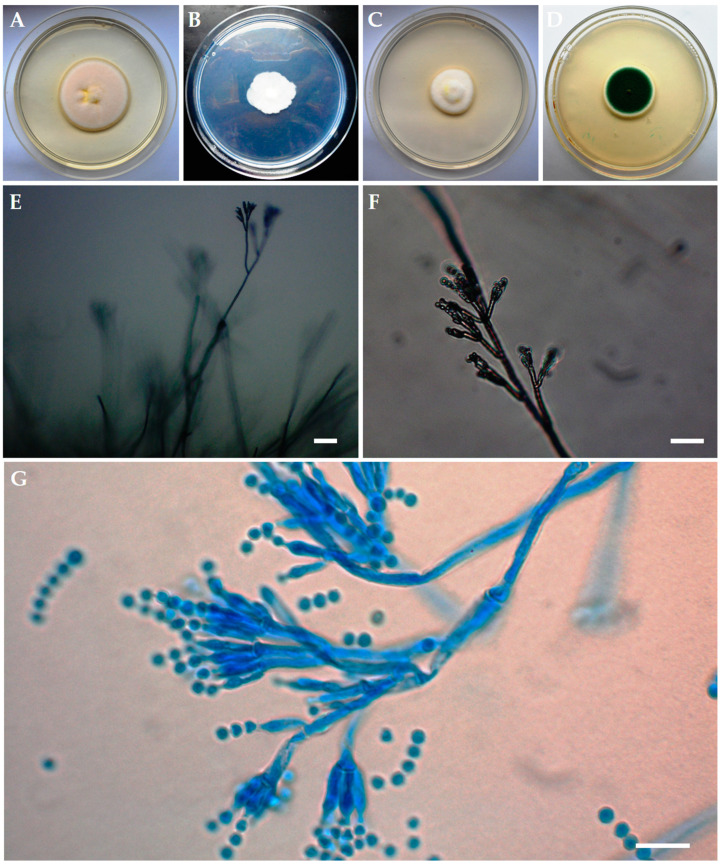
Macroscopic observations of 7-day-old *Penicillium chrysogenum* (**A**). CYA, (**B**). Czapek-Dox agar, (**C**). MEA, and (**D**). PDA and microscopic observations of morphological structures of 2-week-old *P. chrysogenum* (**E**,**F**). Petri dish cultures under the optical microscope with natural arrangement: stipe, branches, ramus, metulae, phialides, conidia, (**G**). Microscopic slide dyed with lactophenol cotton blue: rough branches, ramus and metulae, phialides, conidia) isolated most frequently from the wing membranes of female greater mouse-eared bats (*Myotis myotis*). The size of stipes ranged from 2.5–4 μm, and of phialides ampuliform with a reduced neck from 7–10 to 2–2.5 μm. Conidia were elliptical to subglobose, 3–4 in μm long axis, and smooth. Scale bars: 50 μm (**E**), 20 μm (**F**), 10 μm (**G**,**H**).

*Aspergillus jensenii* and *P. caricicola* were less cultured in the study, and only from one greater mouse-eared bat. Consequently, each of these species accounted for 1.5% of all cultured fungi from all incubation temperatures (Figure 2). On the other hand, the most frequently isolated species from the wing membranes was *P. chrysogenum* (Figure 2 and Figure 3). This fungus constituted 12.3% of all isolated species from all incubation temperatures, and it was isolated from 8 individuals out of 12 tested bats (Figure 2). Color of its colonies ranged from deep white (Czapek-Dox agar) to green (PDA), usually folded center, with broad white margin during the growing period (especially PDA) and in age with overgrowth of white or yellow hyphae (YPG, MEA). The pigmentation on the reverse of the culture media was yellow, with color diffusing somewhat (CYA, MEA, PDA). *Penicillium chrysogenum* formed characteristic microscopic structures such as rough stipes, branches, ramus and metulae (Figure 3).

## 4. Discussions

It is well known that environmental stress is a major determinant in the evolution of living organisms. Therefore, considering the specific conditions prevailing in underground ecosystems as well as the specific physiology and behavior of hibernating bats, these microorganisms have likely adapted and tolerate adverse living conditions [60,61,62,63]. The underground will be mainly a place of occurrence of psychrotolerant fungi because they have better nutritional adaptability compare to psychrophilic fungi or due to horizontal gene transfer from mesophiles [64,65,66]. Thus, bats can be a source of such fungi, which is also illustrated by the results of our research. Moreover, the increase in temperature in underground ecosystems contributes, among other factors, to changes in the fungal communities inhabiting them and may lead to the emergence of new, previously unknown, fungi of mammals, birds and humans [67,68,69,70,71].

In our study, we used PDA because this medium demonstrates comparable efficacy as Sabouraud agar which is most suitable for isolation of a large spectrum of fungal species from biological samples [72,73]. In addition, we used three incubation temperatures (8, 24 and 36 ± 1 °C) for fungal isolations from the samples. In general, most fungi are cultivatable at “room temperature” from 20 to 25 °C. However, psychrophilic and psychrotolerant fungi, e.g., Pd grow better in lower temperatures (12.5 to 15.8 °C for its optimal growth) and these temperatures also correspond to the hibernation place [74]. On the other hand, 36 °C is the temperature oscillating with respect to the body of mammals and thermophilic species [75]. Therefore, we use different temperatures to obtain a broader species spectrum of fungi as well as in the biological context.

Our study allowed us to identify 17 species from two different phyla. Ascomycota being the most numerous with 15 species (88.2%) and Basidiomycota represented by two species (17.8%). According to our knowledge, there are currently no studies on fungi inhabiting the wing membranes of females *M. myotis* during spring emergence from underground hibernation sites. Nevertheless, other researchers confirm that fungi belonging to Ascomycota phylum dominate on wing membranes of bats (*M. sodalis*, *P. subflavus*, *M. septentrionalis* and *M. lucifugus*) hibernating in underground sites [25,32,33]. However, we did not find the fungal species (Pd) on the wing membranes of female *M. myotis* during spring emergence from the “Nietoperek” underground hibernation site. These are unexpected results, because the presence of Pd has been reported in *M. myotis* in every study carried in the “Nietoperek” so far [9,76,77,78]. We think that our sampling targeting bats exiting the bunker either destroyed Pd as the bat body’s temperature becomes elevated, or decreases the number and/or viability of Pd, which confirms other reports on this subject [79,80]. Because the limit temperature for Pd growth is 19.8 °C [74] and usually, during flight, the core body temperature in flying bats may range from 36.9 °C up to 42.1 °C [81,82]. Another reason for the lack of Pd may be the limited number of samples that resulted from permits. This is due to the fact that this species in Poland is strictly protected and requires active protection [83]. Therefore, mycological studies of wintering bats and those flying from underground sites are an important element in the protection of these mammals [5].

The effects of fungi from this study on bat health is unknown. Therefore, we discuss the harmfulness of some of them in the context of people. Additionally, most of *M. myotis* breeding colonies in Poland occur in close proximity to people during the summer, hiding in large attics, church lofts and other buildings [84]. Currently, in Poland, only two breeding colonies use partially underground facilities as breeding places. These are the Studnisko cave in the Sokole Góry nature reserve and the underground fortifications of the “Nietoperek” bat reserve [85,86].

According to Johnson et al. [32] fungi belonging to the *Penicillium* genus accounted for about 13% of all the strains isolated from hibernating bats. This corresponds well which our results, as we confirmed 4 species from this genus *P. brevistipitatum* (found on 5 bats), *P. chrysogenum* (8 bats), *P. citreonigrum* (3 bats) *P. coprophilum* (2 bats). Each one of the tested animals had at least one *Penicillium* species present. The most numerous among them was *P. chrysogenym*, which constituted for 12.3% of all isolated species. This fungal species is commonly recovered in subterranean environments [10,11,12]; however, representing a family pose a potential health hazard, as *Penicillium* airborne, asexual spores are strong human allergens. These fungi are significantly related to an increased incidence of sick building syndrome (SBS) [87]. In specific circumstances, *Penicillium* spp. can pose a serious threat to immunocompromised humans, for example, suffering from AIDS or recovering after transplantations treated with immunosuppressant drugs. For example, *P. marneffei* has been well studied as a cause of severe mycosis in HIV-infected individuals, and *P. chrysogenum* was already reported as the first case of invasive pulmonary mycosis in a lung transplant recipient [88].

In our study, two fungal species belonged to *Aspergillus* (*A. fumigatus* and *A. jensenii*). *Aspergillus* may cause severe skin tineas and human aspergillosis. Furthermore, over the last few decades, filamentous fungi (or moulds) have emerged as a major cause of life-threatening infections in immunocompromised patients [89,90]. Aspergillus fungal spores are commonly found in indoor and outdoor air, including underground ecosystem, and they are also associated with SBS [10,11,12,87,91]. *Aspergillus fumigatus* is probably the “most dangerous” member of *Aspergillus* genus regarding health, this species being classified in risk group 2 regarding pathogen fungi [92].

*Scopulariopsis brevicaulis* is also an interesting species which was cultured during our study. Uusually, it occurs in soil or as a mould on decaying organic matter. However, this species is producing keratinases, proteolytic enzymes, capable of protein degradation. This fungus can cause onychomycosis, keratitis, otomycosis, invasive sinusitis, and prosthetic valve endocarditis. Treatment of *Scopulariopsis* infections is difficult as this genus displays high resistance to a broad spectrum of antifungal agents [93,94].

The *Bartaliniaceae* family was described by Tassi in 1990, and members of the genus have frequently been isolated from the leaves, stems of medicinal plants, or dead aerial spines of *Rosa canina* L. [95,96]. *Bartalinia* spp. is known to produce bioactive compounds eradicating pathogenic bacteria, fungi, and nematodes [97]. *Bartalinia robillardoides*, found on three out of 12 sampled bats is among our most interesting findings as it has been reported to produce taxol, an anticancer drug [98]. In turn, *C. globosum* is a well-known mesophilic mould and it was also isolated during our study. This fungal species is found in a wide range of habitats from forests to mountain soils across various biomes. Its spores can also be found indoors on wooden products, which could explain their presence on bats wing membranes. *Chaetomium globosum* spores are opportunistic agents of mycosis and neurological infections. However, such illnesses occur at low rates [99]. While another isolated species (*C. sphaerospermum*) from *M. myotis* wing membranes is mainly known as a spoilage agent of harvested fruits and vegetables, and it is known as an allergen [100,101].

The next three species (*A. ruber*, *G. aurantiacan* and *A. quadrifidum*) obtained during our research belong to Onygenales order. Nevertheless, only *A. quadrifidum* (Anamorph synonyms: *Trichophyton terrestre*) has medical significance because it may be mildly pathogenic for small mammals but not for human. It is a cosmopolitan, geophilic dermatophyte which can be isolated, among others, from soil and bird feathers [102]. In turn, the last two species obtained during our research are *P. atrogriseum* and *P. caricicola*. Both species are commonly isolated from plants [56,103].

In our study also two species of Basidiomycota were confirmed on *M. myotis* wing membranes—*C. laeve* and *C. ongulense*. *Cylindrobasidium* and *Cystobasidium* genera commonly occur in Poland. They may be found on various types of vegetation, especially on broadleaf trees such as maples (*Acer* spp.), chestnut (*Castanea* spp.), alder (*Alnus* spp.), silver birch (*Betula pendula*), hornbeam (*Carpinus betulus*), hazel (*Corylus* spp.), species belonging to *Rosaceae* family and others. It is far less frequent on coniferous trees however its presence has been confirmed on Scots pine (*Pinus sylvestris*) [104]. Taking into account habitat preferences and the life cycle of bats, it can be assumed that both of those fungi were probably accidentally obtained from the surface of vegetation or bioaerosols around them in the summer season. It is well known that fungal propagation structures, including those inhabiting plants, can be transported by many animals either internally or externally, including bats [9,105,106,107]. Additionally, food and shelter availability are the two most important factors determining the quality of habitats used by bats during breeding season [108] both fungal species can be found in woodlands. Therefore, the vast majority of bats in temperate regions rely on forest habitat for at least part of their life cycle [109].

## 5. Conclusions

This present study reported the colonization of the wing membranes of female greater mouse-eared bat (*M. myotis*) during spring emergence from the “Nietoperek” underground hibernation site by fungi. We believe that our study contributes to a better understanding of the fungal community on the wing membranes of bats. Overall, 17 different fungal species were isolated in the study. The species composition was different between individuals. The wing membranes of individual bats were colonized by 4 to 7 fungal species. The most commonly isolated was the cosmopolitan species *P. chrysogenum*. On the other hand, *A. jensenii* and *P. caricicola* were rarely cultured, and they inhabited the wing membranes of only one individual. Some fungal species may be pathogens of mammals, including bats. However, Pd was not found. Probably, Pd did not survive the elevated body temperature of already active animals as discussed in previous studies and/or too few bats have been tested. Taking into account habitat preferences and life cycle of bats, it can be assumed that some of fungi were accidentally obtained from the surface of vegetation in the during early spring activity. Bats play important functions in ecosystems, and most of them are threatened with extinction. It can therefore be assumed that monitoring the health status and the microbiome of bats seem to be important aspects for the survival of these mammals. Therefore, in the near future, we want to study the mycobiota of other bat species in order to study the physiological microflora including fungi as they could be part of the normal microbiome and contribute to the health of the wing membrane.

## Figures and Tables

**Table 1:** Diversity of fungal species isolated from the wing membranes of 12 female greater mouse-eared bats (*Myotis myotis*).

Micromycetes			*Myotis Myotis* ♀											
Fungal Species	Isolate Number	Accession No. in NCBI Database	1	2	3	4	5	6	7	8	9	10	11	12
*Arachniotus ruber*	UWR_152	MN654344.1					x ^2^		x ^2^		x ^2^			x ^2^
*Arthroderma quadrifidum*	UWR_153	MN654345.1		x ^2^			x ^1^		x ^1^			x ^2^	x ^2^	
*Aspergillus fumigatus*	UWR_154	MN654346.1	x ^3^	x ^3^	x ^3^		x ^2,3^			x ^3^	x ^3^			
*Aspergillus jensenii*	UWR_155	MN654347.1											x ^2^	
*Bartalinia robillardoides*	UWR_156	MN654348.1				x ^2^		x ^2^						x ^2^
*Chaetomium globosum*	UWR_157	MN654349.1	x ^1^									x ^2^	x ^2^	
*Cladosporium sphaerospermum*	UWR_158	MN654350.1		x ^2^		x ^2^				x ^2^	x ^2^	x ^2^		
*Cylindrobasidium laeve*	UWR_159	MN654351.1	x ^2^	x ^2^						x ^2^				x ^2^
*Cystobasidium ongulense*	UWR_160	MN654352.1			x ^1,2^				x ^1,2^					
*Gymnascella aurantiaca*	UWR_151	MN654343.1			x ^1^	x ^1^				x ^1^			x ^1^	
*Pararamichloridium caricicola*	UWR_161	MN654353.1			x ^2^									
*Penicillium brevistipitatum*	UWR_162	MN654354.1			x ^2^			x ^2^			x ^2^		x ^2^	x ^2^
*Penicillium chrysogenum*	UWR_163	MN654355.1	x ^3^			x ^2^	x ^2^		x ^2^	x ^3^		x ^2^	x ^2^	x ^2^
*Penicillium citreonigrum*	UWR_164	MN654356.1			x ^2^			x ^2^				x ^2^		
*Penicillium coprophilum*	UWR_165	MN654357.1	x ^1,2^											x ^2^
*Phialemonium atrogriseum*	UWR_166	MN654358.1		x ^2^		x ^1,2^				x ^2^	x ^2^			
*Scopulariopsis brevicaulis*	UWR_167	MN654359.1	x ^3^			x ^1,2^		x ^1^	x ^1^				x ^1^	

“x” indicates that the fungus was cultured from the samples, ^1^ fungi cultured at 8 ± 1 °C, ^2^ fungi cultured at 24 ± 1 °C, ^3^ fungi cultured at 36 ± 1 °C.
